# Supplementary material for: Are carbon emissions trading and green financial instruments synergistic? -Comprehensive quantitative research based on content analysis
Source: PLoS One. 2024 Mar 7;19(3):e0298601. doi: 10.1371/journal.pone.0298601 (PMC10919601; doi:10.1371/journal.pone.0298601)
Supplement: S1 Data — (ZIP) [file pone.0298601.s001.zip › Data/地级市Z_F工作报告.zip!/╜¡╦╒/│ú╓▌╩╨/└┤╫╘╣1⁄2╓┌║┼í╛┬φ┐╦╩2╛▌═°í┐_2007.docx]

各位代表：

　　现在，我代表常州市人民政府，向大会作工作报告，请予审议，并请市政协各位委员和其他列席人员提出意见。

　　一、2006年工作的基本总结

　　2006年，市政府在中共常州市委的正确领导和市人大、政协的监督支持下，坚持以邓小平理论、“三个代表”重要思想和科学发展观为指导，团结依靠全市人民，以经济建设为中心，开拓创新，务实工作，保持了经济社会发展的良好势头，市十三届人大五次会议确定的各项任务全面完成，总体小康目标如期实现。

　　（一）国民经济较快增长，各项指标完成良好。实现地区生产总值1560亿元，按可比价增长15.2%，按户籍人口计算人均突破5000美元；完成财政总收入277.3亿元，增长25.8%，其中地方一般预算收入突破100亿元，达到118.9亿元，增长25.2%。农业总产值达到109.2亿元，增长4.9%；规模以上工业产值突破3000亿元，实现增加值777.9亿元，增长28%，规模以上工业单位增加值综合能耗下降4%以上；服务业实现增加值548.5亿元，增长15%。城镇居民人均可支配收入达到16649元，增长14.1%；农民人均纯收入超过8000元，增长14%以上；城乡居民存款余额突破1000亿元，比年初增长17.5%；全社会消费品零售总额达到515.8亿元，增长16.2%；城镇登记失业率控制在3.44%。

　　（二）有效投入带动明显，产业结构趋向优化。完成全社会固定资产投资951.6亿元，增长23.6%，其中工业投资562.9亿元，增长22.4%。投资结构进一步优化，高新技术产业投资占工业投资的比重达到14%，服务业投资占全社会固定资产投资的比重达到40.4%。产业层次得到提升，规模以上工业中高新技术产业产值占40.3%，比上年提高3.8个百分点；服务业占地区生产总值的比重提高0.6个百分点；软件和动漫产业发展势头良好，软件企业总数跃居全省第三，常州国家动画产业基地被评为“2006中国十佳最具投资价值创意基地”。

　　（三）利用外资取得突破，对外开放步伐加快。协议注册外资突破30亿美元，达到33亿美元，增长15.6%；实际到帐注册外资突破10亿美元，达到12.5亿美元，增长71.1%，增幅居苏南五市第一。利用外资结构进一步优化。大项目明显增多，新批总投资超3000万美元的达到17个，其中1亿美元左右的有10个；增资扩股和“以民引外”势头良好，227家外资企业新增协议注册外资8.76亿美元，增长48.5%，民营企业协议注册外资占全市总量的22.5%；服务业利用外资比重提升，占实际到帐注册外资总额的29.6%；又有4家世界“500强”企业落户我市，累计达到41家。开发园区建设取得新进展。省级以上开发区完成基础设施投入74.7亿元，增长49.4%；口岸功能进一步增强，出口加工区封关运作，海关、国检武进办事处和录安洲港区码头一期工程开工建设。外贸进出口总额突破100亿美元，达到104.4亿美元，其中出口78.9亿美元，增长28.8%。外经营业额达到2.4亿美元，增长20%。新批境外投资项目27个。3家企业在境外上市，天合光能成为国内第三家在纽约证券交易所挂牌交易的民营企业。成功举办中国常州科技经贸洽谈会和国际友好城市合作与发展论坛，对外合作与交流得到加强。

　　（四）科技创新不断深化，企业活力普遍增强。全社会研发经费占生产总值的比重达到1.52%。产学研合作进一步加强，成立了中科院常州先进制造技术研发与产业化中心，并启动建设智能机械等7个分中心，成立南京大学常州高新技术研究院，建成产学研合作基地和示范企业15家。公共创新平台建设取得新进展，市技术产权交易中心成立并投入运行，新增国家级企业技术中心1家、省级工程技术研究中心3家、博士后科研工作站2家。企业创新能力不断增强，承担省级以上科技创新项目191项，获国家科技进步二等奖2项、省科技进步奖11项，引进各类人才1.85万名，获得授权专利1553件，新增省级高新技术企业63家、高新技术产品190只，新增中国名牌产品8只、中国驰名商标4件。企业活力进一步增强。完成10家国有企业的国有产（股）权转让和4家事业单位的改制；营业收入超10亿元、利税超亿元的企业比上年分别增加12家和7家；新增私营企业8000户、注册资本140亿元，民营经济占全市经济总量的比重超过60%。

　　（五）城市建设力度加大，人居环境不断改善。完成新一轮城市总体规划的25个配套专项规划和一批重要地区的城市设计，实现中心城区控制性详细规划全覆盖。初步完成京沪高速铁路、城际轨道交通常州段的线网规划。宁常高速、扬溧高速、运河南移工程加速推进，104国道、241省道等6条干线公路建成通车。长江路、常焦路、常金路和青洋路全线通车，城市快速高架路一期工程开工建设。启动建设武南河拓浚工程和城市生活垃圾焚烧发电项目。“八路八口”绿化工程全面完成，红梅公园扩建提升后免费开放，建成了市民广场等一批公共绿地，新增绿地1600公顷，城镇人均公共绿地达到9.2平方米，建成区绿化覆盖率达到40.6%。武进建成全国生态示范区。实施“清水工程”，完成中心城区23条河道的整治。开展了主要道路市容环境综合整治，城市面貌有所改观。全面整合市区公交资源，新增6条公交线路和5个回车场。城市管理不断创新，“条块结合、以区为主、重心下移、属地管理”的城市管理新体制基本形成。

　　（六）社会事业协调发展，政治社会和谐稳定。坚持教育优先发展。全免了城乡义务教育阶段的学杂费。全市外来务工人员的子女全部入学就读，其中77%进入公办学校。高考本科录取率、万人进线率和职校单招成绩继续名列全省第一，江苏工业学院等5所高校顺利通过教育部教学工作评估。医疗卫生事业健康发展。全市新型农村合作医疗参保率达到98%，城市社区卫生服务覆盖率达到90%，市医疗急救中心主楼等公共卫生设施建成使用。人口继续保持低增长水平。体育事业取得新成绩，申办省第十七届运动会圆满成功，体育中心二期等重点场馆建设进展顺利。文化广电事业又有新进步。常州博物馆、规划馆、书画院新馆、文艺学校新校区相继建成，张太雷纪念馆、金坛三星村遗址成为全国文物保护单位，金坛市建成全国优秀旅游城市。社会保障事业进一步发展。城镇净增就业5.1万人，下岗失业再就业2.6万人，农村劳动力转移就业5万人；参加养老保险净增10万人，养老、医疗和失业三大保险综合覆盖率达到96.4%，大幅度提高了企业退休人员的养老金标准，实施了城乡低保的提标扩面；慈善救助事业蓬勃发展，全市慈善基金总量突破10亿元，居全国省辖市第一，发放慈善救助资金4200多万元；扩大了廉租房、经济适用房和住房公积金贷款的受益面。年初确定的实事项目，除青枫公园二期调整实施外全部完成。精神文明和民主法制建设进一步加强，启动实施“五五”普法教育，深入开展“平安常州”建设，社会秩序稳定良好。安全生产体制机制不断完善，有效防止了重特大事故的发生。金融生态安全区创建和以食品药品安全为重点的市场秩序整顿工作取得实效。人防工作、国防动员和民兵预备役建设取得新进展，连续三次获得“全国双拥模范城”称号。与此同时，外事、侨务、对台事务、档案、地方志、防震减灾、民族宗教、老龄、妇女儿童、关心下一代等工作都取得了新成绩。

　　（七）“三农”工作成效明显，新农村建设有序推进。粮食生产水平稳步提高，水稻单产和机械化插秧率名列全省第一。农业结构调整步伐加快，高效农业面积增加6.8万亩，新增5项省级农业地方标准、2个省级农业标准化示范区和17个无公害产品产地，新批5个国家级农业旅游示范点。“三资”开发农业的投入达到24亿元。农村改革深入推进，完成264个村股份合作制改革，新成立各类农民专业合作经济组织44家，组建成立7家土地股份合作社。社会主义新农村建设取得良好开局，启动建设8个示范镇、20个示范村和58个居住示范点，新建、改建农村公路431公里。茅山老区扶贫帮困取得实效，落实各类扶持资金3400万元，实施了一批基础设施项目和农民致富项目。

　　（八）自身建设得到加强，工作效率有所提高。自觉接受市人大、政协和各民主党派的监督，474件建议和提案全部办结；完善部门预算制度，积极推进政府收支分类、国库集中收付和非税收入等改革，全面开展政府投资项目跟踪审计，勤政廉政建设取得新的成效。行政审批制度改革进一步深化，许可类、非许可类行政审批工作日分别压缩了47%和46%，又有21个项目实行即时办理，初步建成网上审批系统。开展了“创优环境兴常州、改进服务促发展”专项行动，加大落实和督查力度，企业和人民群众对发展环境的满意度有了提高。我市名列“全国城市创新环境评价”第七位，跻身“2006中国投资环境百佳城市”行列。

　　各位代表，去年我市经济社会发展所取得的一切成绩，都是在中共常州市委的正确领导下，全市人民团结奋斗、共同努力的结果。在此，我代表市人民政府，向全市人民，向市人大代表、政协委员、各民主党派、工商联、各人民团体、离退休老同志、无党派及各界人士，向驻常部队指战员、武警官兵和部、省驻常单位，向所有关心、支持常州建设和发展的海内外朋友们，表示衷心的感谢！

　　在肯定成绩的同时，我们也清醒地看到工作中存在的问题与不足。主要表现在：虽然有效投入力度在加大，但结构调整进展比较缓慢，服务业的比重还偏低，产业优势还不明显；虽然利用外资快速增长，但外资规模还不大，特别是带动产业结构优化升级的超亿美元大项目还太少，利用外资的基础还比较脆弱；虽然全市总体达到全面小康，但人均数并不代表大多数，一些地区特别是茅山老区的农民收入还不多，一些群众的生活还比较困难；虽然各地科学发展的意识在不断增强，但资源和环境的矛盾依然十分突出，特别是一些地区环境污染引发的社会矛盾时有发生；虽然政府的职能在转变，但机关作风和效率还适应不了形势的变化和人民群众的期待，勤政廉政建设还需进一步加强。这些问题都有待在今后的工作中加以改进。

　　二、2007年目标任务和重点工作

　　今年政府工作总的要求是：以邓小平理论、“三个代表”重要思想和科学发展观为指导，认真贯彻落实市第十次党代会精神，坚持率先发展、科学发展、和谐发展，全面推进创新型城市、生态城市、文明城市、新农村和富裕常州“五大建设”，巩固和提升全面小康建设成果，实现以县为单位的全面小康，努力开创常州经济社会又好又快发展的新局面。

　　今年经济社会发展的主要目标是：

　　——地区生产总值增长15%以上；

　　——地方一般预算收入增长15%以上；

　　——全社会固定资产投资增长20%以上；

　　——实际到账注册外资增长20%以上；

　　——进出口总额增长20%以上，其中出口增长25%以上；

　　——全社会消费品零售总额增长15%以上；

　　——城镇居民人均可支配收入增长12%以上；

　　——农民人均纯收入增长10%以上；

　　——城镇登记失业率控制在4%以内；

　　——全社会研究和开发经费占生产总值的比重在1.6%以上；

　　——单位生产总值能耗下降4.5%以上；

　　——环境质量综合指数得分在80分以上；

　　——废水化学需氧量减排4%、二氧化硫含量减排2.7%。

　　为实现上述目标，我们将重点抓好以下八项工作：

　　（一）转变增长方式，加快结构调整，着力增创经济发展新优势

　　突破发展装备制造业。按照建设装备制造业名城的目标，在政策导向、资金扶持、要素供给等方面，重点支持初具规模和发展潜力大的装备制造产业。充分利用我市输变电产业优势，着力推进超大型变压器、特种电缆等项目建设，提升输变电产业的配套性和竞争力；加快轻轨、高速铁路配套系统项目建设，扩大轨道交通产业的市场份额；大力发展多功能挖掘装载机等工程机械，努力成为全国工程机械重要生产基地；重点发展环保节能型多缸柴油机和农机终端产品，加快形成农业机械产业集群；加大新型数控机床开发力度，提高基础装备产业的现代化水平。

　　大力发展现代服务业。不断提高服务业的辐射扩张能力和对制造业的支撑水平，重点发展现代物流、研发设计等生产性服务业，大力培育信息中介、技术咨询、金融保险等新兴服务业。加快推进常州高新区综合物流园区等50个现代服务业项目建设，扶持横林地板市场、湖塘纺织城等服务业集聚区的发展。着力引进新型股份制商业银行，促进外资银行在常拓展业务。

　　积极培育新兴产业。加快发展新材料、新能源和生物医药等新兴产业，着力推进天合光能太阳能电池、常牵兆瓦风机等一批重点项目建设。大力培育动漫和软件产业，加快西太湖动漫基地的规划建设，发挥常州国家动画产业基地的集聚功能，构筑动漫生产、销售及衍生产业链；加快常州软件园二期建设，扩大软件外包业务，培育知名软件品牌，力争软件业销售额实现翻番。

　　改造提升传统产业。坚持信息化带动，积极实施50个产业链长、成长性好的制造业升级项目。运用高新技术和先进适用技术，加快纺织、服装等行业的技术改造，淘汰落后生产工艺和设备，提升传统产业的科技含量和附加值。鼓励企业通过搬迁改造实施技改扩能，通过集聚发展和资产重组实现规模扩张。全面开展化工行业的专项整治，引导化工、印染企业走集约发展之路。

　　开展“重大项目推进年”活动。组织实施一批关联度大、带动力强的大项目，一批科技含量高、资源消耗低、环境污染少、能够优化产业结构的好项目。建立并落实好各级领导对重点成长型企业、工业重点项目和重大外资项目的挂钩服务制度。积极引导民营企业创新发展理念，加大投入力度，增强发展后劲。围绕项目推进，搞好协调服务，破解发展难题，强化督查考核，营造全市上下共同推进项目建设的良好氛围。

　　（二）加快科技创新，做强做大企业，努力增强区域经济竞争力

　　实施“五大工程”，推进科技创新。一是抓好创新企业培育工程，力争新增创新型试点企业和省级高新技术企业各30家以上，高新技术产业产值占规模以上工业的比重提高2个百分点。二是抓好创新项目带动工程，实施7个市级科技专项和50个重点创新项目。三是抓好创新平台建设工程，完善高新区创业中心等4个国家级创业平台功能，新增15个重点科技创新平台；发挥科教城的集聚功能，推进中科院常州先进制造技术研发与产业化中心建设，深化与清华、南大、东大等高校的产学研合作，新增20个中试基地和合作示范企业。四是抓好创新人才集聚工程，培养、引进20-30名高层次创新人才，力争引进3-5个海外归国的领军型创业人才及其团队。五是抓好创新环境优化工程，进一步整合各种资源，发挥各类优惠政策和扶持资金的导向与激励作用。

　　实施强企战略，壮大规模经济。深入开展“创星争先、做强做大”活动，鼓励企业做精、做专、做特主营产业，提升核心竞争力。加大品牌建设力度，力争新增中国驰名商标2件、中国名牌产品6只以上，培育40个产业链长、发展前景好的特色产品集群。引导企业实施资本经营，通过收购、兼并、上市等措施，实现裂变发展，力争有3家以上企业在境内外上市。与此同时，继续深化国有企业改革，进一步改进对中小企业的服务，促进面广量大的中小企业快速健康发展。

　　提升县区发展水平，增强区域经济竞争力。加快工业化、城市化进程，充分发挥县域经济发展的巨大潜力。金坛要做强做大盐化工、氟化工、光伏和服装等特色产业。溧阳要做优天目湖品牌，做强特种电缆、变压器、旅游等特色产业。武进要大力发展机械装备、微电子等优势产业，做强做优现代服务业，成为全市现代制造业和服务业的重要基地。新北要依托国家级高新区，做强做大新材料、新能源、电子信息、软件及物流业等新兴产业，成为全市现代制造业的重要基地和高新技术发展的主阵地。天宁、钟楼、戚区要加快城乡一体化进程，以现代服务业和都市工业为重点，全面提升经济发展水平。

　　（三）扩大对外开放，突破利用外资，自觉融入国际经济大循环

　　量质并举，促进利用外资再上新台阶。瞄准世界500强、跨国公司和国际行业龙头企业，大力引进产业带动强、科技含量高、投资强度大、资源消耗低的项目，力争引进投资超亿美元的项目10个以上。积极实施产业链招商，促进外资向产业上下游延伸，吸引外资投向装备制造产业、现代服务业、新兴产业和现代农业。加强对外资企业的服务，促进外资企业增资扩股和“以外引外”。充分发挥民营企业的引资载体功能，积极推进“以民引外”。进一步强化各级招商考核激励机制，健全境外招商代理网络，形成一支善攻坚、素质高的专业招商队伍。精心组织好境内外重大招商活动，举办好2007中国常州科技经贸洽谈会。与此同时，坚持招商引资与招才引智相结合，全方位提高我市的开放水平。

　　提升园区建设水平，增强项目承载能力。常州高新区要进一步整合土地资源，提高大项目的承载能力；推进电子工业园、录安洲港区建设和沿江开发，进一步完善出口加工区功能。武进高新区要进一步加快基础设施建设，着力推进海关、国检、保税物流中心等配套功能建设，努力构筑科技创业园、模具产业园等园区特色。其他各省级开发区的基础设施建设投入要确保增长30%以上，实际利用外资增长50%以上。各乡镇工业集中区要结合新农村建设，大力推进污水集中处理、水电气等配套设施建设，为项目进区创造良好条件。

　　实施“走出去”战略，进一步提高外贸外经水平。推进“科技兴贸”和“以质取胜”战略，支持出口企业开发拥有自主知识产权和核心技术的产品，进一步提高机电和高新技术产品的出口份额，新增3个国家、省级重点培育发展的出口名牌。优化进出口结构，扩大先进技术装备、关键零部件和短缺资源的进口。支持有条件的企业开展境外投资和跨国经营，开发利用国外能源和矿产资源。同时，积极扩大对外交往，创新对外宣传，不断提升常州在国际上的知名度。

　　（四）落实富民优先，着力发展现代农业，加快建设社会主义新农村

　　大力发展现代农业，促进农民持续增收。坚持科技兴农和“三资”开发农业，促进农业结构调整优化。大力发展高效农业，加快建设长荡湖等20个示范基地和园区，重点发展特色畜禽、特种水产、花卉苗木等优势产业。加快发展品牌农业，放大天目湖等知名品牌效应，扶持具有鲜明特色的“十镇百村”，逐步形成“一镇一业”、“一村一品”的发展格局。着力发展休闲观光农业，新增国家级农业旅游示范点4个，接待游客突破20万人次。积极发展规模农业，扶持产供销、贸工农、种养加一体化的专业合作社和龙头企业，建设好凌家塘等一批农副产品市场，形成规模效应显著的农业生产经营体系。

　　深化农村各项改革，完善支农惠农政策。扶持粮食生产，加大对农民的直接补贴力度，切实保护种粮农民利益。加强涉农收费项目监管，有效防止农民负担反弹。深入推进和规范农村集体经济股份合作制改革，符合条件的村组年内要全面完成；引导农民专业合作社向股份型、紧密型、龙头型方向发展；积极推进以土地承包经营权入股、集体资金参股为主要形式的土地股份合作制改革，逐步形成种养大户、普通农户和集体经济多赢的发展机制，确保农民长效增收。

　　继续开展茅山老区帮扶活动，积极实施农村新五件实事。深入开展党政机关与贫困镇村结对帮扶活动，多渠道筹集帮扶资金，落实帮扶项目，推进基础设施建设，切实加快老区脱贫致富步伐。深入实施新农村建设三年行动纲要，扎实推进农村新五件实事：（1）农村道路通达工程，新建和改造农村等级公路420公里；（2）农民教育培训工程，完成农村劳动力转移培训2.5万名、农业实用技术培训6万人次；（3）农村环境整治工程，开展“三清一绿”活动，实施250个行政村1000万立方米的河塘疏浚；（4）农村文化建设工程，建设一批农村文化馆站和农家书屋，实施有线电视“村村通”工程；（5）农民健康工程，新建一批医疗站点，完善农村卫生服务网络，逐步实现农村医疗站点全覆盖。

　　（五）贯彻以民为本，加快城乡建设，进一步优化人居环境

　　坚持“民本、文化、现代、生态”的理念，按照“一年一个样，三年大变样，五年展辉煌”的要求，加快城乡现代化建设进程。

　　拉开城市框架。突出规划的龙头作用，完善综合交通体系规划，加快完成中心城、南北新城“三城”近期建设规划，开展城市东西部发展的规划研究。加快建设以高速公路、干线公路为主的公路网络，建成宁常、扬溧高速常州段，开工建设西绕城高速公路，完成运河南移工程，加快建设高架路，建成健身北路延伸段，延伸东方大道、竹林北路，新建飞龙东西路、玉龙路等道路。

　　建设南北新城。加快南北一体化进程，高标准建设武进、新北两个新城区，推进城市要素与资源的合理配置和集约利用。武进要加快建设5.6平方公里核心区，加快建成武进客运中心、泰富城等项目，积极推进西太湖生态休闲城和太湖湾旅游度假区建设，重点建好滨湖公园等项目。新北区要加快以中华恐龙园周边、通江南路（原常澄路）两侧为重点的核心区建设，推进新北医院等社会事业项目建设，增强商业、旅游设施的辐射功能，进一步提升商贸承载能力和公共服务水平。

　　实施重点工程。精心组织实施城建十大工程：（1）城市高架路建设工程，完成总长29公里一期工程量的70%，2008年上半年建成通车；（2）运河南移工程，年底完工通航，11座跨河大桥全部竣工；（3）“八路八口四河”绿化工程，对宁常、宁杭、扬溧高速等八条公路及其八个道口，以及新运河、长江常州段等四条河道实施绿化，新增绿地2000公顷；（4）城市主要道路环境综合整治和美化亮化工程，提升通江南路、和平南路（原常武路）的整体水平，对同济桥周边、晋陵路、关河西路、广化街等道路环境进行整治；（5）高速公路和城市道路建设工程，建成2条高速公路，新开工1条高速公路和8条城市道路；（6）常州大剧院、体育中心及会展中心建设工程，年内主体完工；（7）太湖湾和西太湖开发建设工程，太湖湾主要景点5月份建成开放，西太湖完成环湖大道等主要道路建设；（8）西瀛里商业中心建设工程，完成20万平方米建设任务，确保年内基本建成并部分开业；（9）兰陵片老城区改造工程，年内完成规划等前期工作和15万平方米拆迁，3年内基本完成改造任务；（10）录安洲开发工程，建成2个万吨级通用码头，开工建设2个万吨级石化码头。

　　落实长效管理。进一步完善城市管理新体制，形成职责明确、齐抓共管、运转高效的长效管理机制。全面落实13项长效管理制度，深入开展“规范交通管理，解决‘三难一乱’”专项行动，切实解决“行驶难、停车难、乘公交难和交通秩序乱”的问题。

　　统筹城乡建设。加快编制中心镇、工业集中区布局规划，实施“城中村”改造，推进示范镇、示范村和居住示范点建设。完善工业集中区基础设施功能，促进乡镇企业集约发展。加大对农村基础设施的投入，加快农田水利、电力通信和村容村貌建设，着力解决人畜饮水、垃圾污染等突出问题。大力发展农村各项社会事业，进一步改善农民生产生活条件。

　　（六）加强环境保护，降低资源消耗，实现经济社会可持续发展

　　坚持“三调四严”，实施“六治一绿”，切实保护和修复环境，推进生态城市建设。落实环保优先方针，调整产业结构、企业布局和发展思路，严格环保的准入门槛、执行标准、监督执法和考核评价。着力推进“治水、治气、治音、治土、治村、治废”和“全民造绿”工程。采取工业治污、生活截污、河道清淤、循环活水等措施，全面提升城乡水环境质量。推进“禁燃区”建设，积极实施脱硫工程，加强机动车尾气防治，严格控制大气污染物排放。大规模开展绿化造林，建成10个国家级环境优美镇、30个省级生态村和100个生态园林单位，建成区绿化覆盖率提高1.4个百分点，确保建成国家园林城市。加快环保基础设施建设，开工建设江边污水处理厂二期工程，新建和扩建溧阳、金坛生活垃圾卫生填埋场，建设10个乡镇污水处理厂。

　　坚持节约优先，发展循环经济，降低资源消耗。按照“减量化、再利用、资源化”原则，进一步推进循环经济示范点建设，逐步构建覆盖企业、产业、园区的循环经济体系，实现资源的循环和高效利用。大力推行清洁生产，对重点行业实行强制性清洁生产审核。建立节能降耗的指标和考核体系，重点抓好冶金、水泥、化工等行业的节能降耗工作，逐步淘汰高耗能、低效益产业。

　　集约利用土地，提高投入产出强度，实现可持续发展。把投入强度列为工业用地的前置条件，省级以上开发区新批项目每亩投入不低于250万元，乡镇工业集中区不低于160万元，中小企业进入标准厂房。鼓励园区内企业利用现有土地，扩产增容，努力提高单位面积产出水平。盘活现有土地存量，加大土地复垦开发力度，努力扩大增量，保持耕地占补平衡。

　　（七）发展社会事业，增加公共产品，全面推进和谐常州建设

　　大力发展科教、卫生、文化、体育等社会事业。积极实施素质教育，大力开展农村“合格学校”建设，努力推进城乡基础教育均衡发展和区域教育现代化。推进新一轮职业教育布局调整，进一步提升高等教育办学水平，培养更多的经济、社会适用人才。启动实施公民科学素质行动计划，提高全民科学素养。大力发展社区卫生服务，城市和农村社区卫生服务覆盖率分别达到95%和100%，逐步实现“小病在社区、大病到医院、康复回社区”的目标；加强公共卫生体系建设，增强公共卫生突发事件的应急能力。改革文化体制，整合文化广电资源，大力发展文化产业，争创一批文艺精品。加强对古运河的保护与开发，完成青果巷历史文化街区修复保护规划，全面推进前后北岸历史文化街区等文物修缮工程，保护好百年以上的古典园林并完成1-2家修复保护方案的论证。以筹办第17届省运会为契机，努力提升竞技体育水平，深入开展全民健身运动，积极发展体育产业。与此同时，积极有序推进事业单位分类改革，继续做好外事、侨务、对台事务、地方志、民族宗教、防震减灾、关心下一代、人口与计划生育等工作，支持发展妇女儿童、老龄和残疾人事业。

　　优化财政支出结构，加快公用事业项目建设。增加对教育、卫生和环保等公用事业的投入。加快建设科教城产学研重点工程和武进、新北及刘国钧职教中心；开工建设市二院（阳湖医院）、残疾人康复中心，完成市应急中心、疾控中心、市一院门急诊大楼等重点项目；启动市图书馆改扩建工程；建设一批群众健身场馆设施。

　　扩大就业途径，完善社会保障。建立和完善人力资源市场体系，形成劳动者自主就业、市场调节就业、政府促进就业的新机制。加大就业培训力度，重视解决零就业家庭、困难群体、低龄失业和“4050”人员的就业问题。健全多层次的社会保障体系，着力扩大各类企业特别是灵活就业人员的社保覆盖面，完善困难和破产企业职工进入医保的办法。健全社会救助体系，加强社会福利和救灾救济等工作。大力发展慈善事业，充分发挥慈善基金在济困、助学等方面的积极作用。

　　解决突出问题，维护社会稳定。全面推行企业职工工资集体协商制度和劳动合同制度，建立与企业效益相适应的工资收入增长机制。维护征地、拆迁政策的统一、公平和严肃性，强化属地政府的安置补偿责任，保障被征地农民的生产生活，维护群众正当权益。依法惩处各类违法犯罪行为，有效维护社会治安。整顿规范市场经济秩序，全面推行安全生产责任制，确保人民生命财产安全。健全应对各类突发性事件的工作机制，提高公共安全应急处置能力。

　　加强精神文明和民主法治建设。进一步加强公民特别是未成年人思想道德教育，加大文明城区、文明社区的创建力度。继续做好国防动员、民兵预备役、拥军优属和优抚安置等工作。整合市、区、街道、社区的社会管理和服务资源，形成党委领导、政府负责、社会协同、公众参与的社会管理格局。深入推进“五五”普法，开展“民主法制村（社区）”建设，提高法律援助和服务水平。自觉接受市人大的法律监督、工作监督和市政协的民主监督，主动听取民主党派、工商联、无党派人士和各人民团体的意见和建议，认真办理人大代表、政协委员的议案、建议和提案。

　　（八）立足人民得益，注重改善民生，继续为民办好十件实事

　　按照内容更实、惠及面更广的要求，今年将办好以下十件实事：（1）扩大劳动就业。城镇就业净增4万人以上，农村劳动力转移就业2万人以上，援助困难人员就业3000人，下岗失业人员再就业1.8万人。（2）提升救助水平。城乡低保月标准提高20元以上，年底增发一个月全额低保金，并对低保边缘困难群众年底按户发放700元的一次性救济金。继续对患有恶性肿瘤、白血病、尿毒症等三类重大疾病的市区低保对象、低保边缘困难群众和个人支付医疗费用超过家庭年收入3倍以上的一般群众，给予一次性大病救助。新增孤寡老人供养床位2000张以上，农村“五保”老人集中供养能力达到80%。（3）完善社会保障。养老、医疗保险分别净增参保3万人以上，失业保险净增参保2万人以上。建立惠及城镇老年居民和少年儿童的医疗保险制度；完善新型农村合作医疗保险，市、辖市（区）财政的年补贴标准每人再提高20元。（4）改造市区菜市场。年内实施原址改造12个、搬迁移建13个，完成内部提升24个，用三年时间全面完成改造任务。（5）继续实施“清水工程”。对北塘河等19条河浜进行整治，全面完成中心城区主要河道的整治任务，使市区水环境得到明显好转。（6）建设“五园三绿地”。新建青枫公园二期、荷园、武进新天地公园、淹城公园一期和扩建敞开花溪公园等5个免费公园，建设青山桥、南市河和马公桥等3块公共绿地。（7）优先发展公交。分阶段降低公交票价，除少数长线公交，空调车票价由原来的2元降至1元，持IC卡六折、学生卡三折、老人卡两折乘车，残疾人等对象继续享有免费优惠；新增公交车215辆，并提高公交车辆档次；开辟公交专用车道，规划建设新北区至大学城的大容量快速公交系统，切实解决人民群众“出行难”问题。（8）实施街巷里弄整治。对老城区街巷里弄实施改造整治，年内完成280条。（9）改善低收入群体居住条件。制定实施住房保障五年规划，新建蓝天新苑、新都市花园一期、飞龙新苑等经济适用房50万平方米，提供经济适用房和廉租房2000套，发放住房公积金低息贷款16亿元；完成5个共20万平方米的老住宅小区综合整治。（10）继续开展“百村帮扶、万户解困”活动。多措并举，力争两年内使茅山老区经济薄弱村和低收入农户实现“三通、五确保、一有”目标，即：通有线电视、电话、班车，确保出行方便、饮水安全、医疗保障、无草危房、农业增效，集体经济有稳定来源。

　　三、建设服务型政府

　　围绕政府职能转换，创新行政服务理念，规范行政审批行为，提高行政服务效能。具体做到：民本、诚信、规范、高效、清廉。

　　民本，就是贯彻以人为本的原则。把“一切为了人、依靠一切人”作为政府工作的出发点和落脚点。全心全意为人民谋利益，把人民满意不满意、赞成不赞成、拥护不拥护作为衡量政府工作的标准和政府决策的依据，把人民群众的反映作为改进政府工作的第一信号。凡是人民群众拥护、赞成的事多做快做，凡是人民群众反对和厌恶的事坚决不做。要为人民群众多办好事实事，帮助他们解决生产生活中的实际困难，让人民群众更多地分享到改革发展的成果。

　　诚信，就是建设诚信政府。诚信社会首先要看政府的公信力，政府诚信是社会诚信的导向和榜样。全市各级政府都要做到言必行、行必果。凡是在人代会上向全市人民作出的报告，凡是政府常务会议和市长办公会议决定的事项，都要不折不扣地办到做到。凡是对企业、对群众作出的承诺，都要一诺千金，能够做到十分，先承诺七分，承诺了七分，就要努力争取做到十分。每个政府组成人员和公务员都要从我做起，以自己的实际行动取信于民，维护政府的诚信形象。

　　规范，就是坚持依法行政、规范运作。坚持法律至上，把政府的各项工作严格规范在法律许可的范围之内，严格按照法定的权限和程序行使权力、履行职责。政府的各项工作都要讲法制、讲规范、讲程序，落实好行政执法责任制。要提高政府工作的透明度，政府的一切从政行为都可以也应该向市民公开，广泛听取市民意见，自觉接受人民监督。

　　高效，就是做到简化、明确、快速、见效。简化，就是要简化办事程序，缩短办事时限，把复杂问题简单化，要多讲群众听得懂的“地方话”，多给企业解决问题的办法。明确，就是对受理事项要有明确的态度、条件、手续和期限，对暂不符合要求的要一次性告知所缺条件。快速，就是对企业和群众需要帮助解决的问题，要“马上办”，而且要想办法、出主意，千方百计帮助他们“办上马”。见效，就是要做到今日事今日毕、明日事提前做，争取一个工作日能完成两个工作日的任务，一年能够做完两年的事情。

　　清廉，就是清正廉洁，既要干事又要干净。全面落实廉政建设责任制，健全完善惩治和预防腐败的各项制度，坚决纠正损害群众利益的不正之风，着力整治商业贿赂，加大对违纪违法案件的查处力度。加强行政监察、财政监督和审计监督，着力建设节约型机关，严格执行部门预算、政府采购和建设项目公开招投标等各项制度，努力从源头上、制度上预防腐败。

　　各位代表！形势催人奋进，任务光荣艰巨。让我们在中共常州市委的领导下，锐意进取，扎实工作，开拓创新，再展宏图，为实现富民强市的宏伟目标而努力奋斗！
